# The In Vitro Anti-Inflammatory Activities of Galangin and Quercetin towards the LPS-Injured Rat Intestinal Epithelial (IEC-6) Cells as Affected by Heat Treatment

**DOI:** 10.3390/molecules26247495

**Published:** 2021-12-10

**Authors:** Shi-Qing Cai, Qiang Zhang, Xin-Huai Zhao, Jia Shi

**Affiliations:** 1 Key Laboratory of Dairy Science, Ministry of Education, Northeast Agricultural University, Harbin 150030, China; lukecaisq@163.com; 2 School of Biology and Food Engineering, Guangdong University of Petrochemical Technology, Maoming 525000, China; zhangqiang@gdupt.edu.cn; 3 Research Centre of Food Nutrition and Human Healthcare, Guangdong University of Petrochemical Technology, Maoming 525000, China; 4 Maoming Branch, Guangdong Laboratory for Lingnan Modern Agriculture, Guangdong University of Petrochemical Technology, Maoming 525000, China

**Keywords:** IEC-6 cells, flavonol, lipopolysaccharide, anti-inflammatory effect, TLR4/NF-κB signaling pathway, molecular docking

## Abstract

Flavonols possess several beneficial bioactivities in vitro and in vivo. In this study, two flavonols galangin and quercetin with or without heat treatment (100 °C for 15–30 min) were assessed for their anti-inflammatory activities in lipopolysaccharide (LPS)-stimulated rat intestinal epithelial (IEC-6) cells and whether the heat treatment caused activity changes. The flavonol dosages of 2.5–20 μmol/L had no cytotoxicity on the cells but could enhance cell viability (especially using 5 μmol/L flavonol dosage). The flavonols could decrease the production of prostaglandin E_2_ and three pro-inflammatory cytokines interleukin-1β (IL-1β), IL-6, and tumor necrosis factor-α, and simultaneously promote the production of two anti-inflammatory cytokines IL-10 and transforming growth factor-β. The Western-blot results verified that the flavonols could suppress the LPS-induced expression of TLR4 and phosphorylated IκBα and p65, while the molecular docking results also illustrated that the flavonols could bind with TLR4 and NF-κB to yield energy decreases of −(21.9–28.6) kJ/mol. Furthermore, an inhibitor BAY 11-7082 blocked the NF-κB signaling pathway by inhibiting the expression of phosphorylated IκBα/p65 and thus mediated the production of IL-6/IL-10 as the flavonols did, which confirmed the assessed anti-inflammatory effect of the flavonols. Consistently, galangin had higher anti-inflammatory activity than quercetin, while the heated flavonols (especially those with longer heat time) were less active than the unheated counterparts to exert these target anti-inflammatory effects. It is highlighted that the flavonols could antagonize the LPS-caused IEC-6 cells inflammation via suppressing TLR4/NF-κB activation, but heat treatment of the flavonols led to reduced anti-inflammatory efficacy.

## 1. Introduction

The intestinal epithelium, as an important element of the intestinal barrier, can protect the body from the entrance of various harmful substances including bacteria and endotoxins. Lipopolysaccharide (LPS) is one of the most common bacterial toxins that are found in foods. LPS comes from the outer membrane of the Gram-negative bacteria and is well-known for its ability to induce food poisoning; meanwhile, LPS also can impair the barrier function of the epithelial cells and induce cellular inflammation simultaneously [1]. It is known now that the damaged intestinal lumen leads to an easier entrance of toxins into the body circulation, which thereby might promote the release of inflammatory factors, induce inflammation and abnormal immune response, and even cause increased susceptibility to inflammatory diseases like food allergy, inflammatory bowel disease (IBD) and celiac disease. Generally, IBD is a chronic inflammatory disease of the gastrointestinal tract including Crohn’s disease (CD) and ulcerative colitis (UC), while its incidence and prevalence are increasing worldwide [2]. Thus, it is vital for us to maintain normal intestinal barrier function and alleviate the LPS-induced intestinal inflammation. In general, the pharmacological approaches might induce some side effects during their long-term use in the body [3]. Interestingly, there is growing evidence demonstrating the beneficial effects of natural polyphenols in the body, including their barrier protection and intestinal anti-inflammatory function.

Natural polyphenols are widely distributed throughout the vegetable and fruit kingdom, which are part of our daily diets [4]. Polyphenols are rich in the seeds, roots, tree bark, and leaves of various plants [5], and are regarded to have a wide range of biofunctions that are beneficial to the body [6]. For example, a flavone compound apigenin was found to have an anti-cancer effect on the colon carcinoma (HCT-116) cells by inhibiting cell proliferation, disturbing cell cycle progression, destroying the mitochondrial membrane, and inducing cell apoptosis [7], while two flavonol compounds quercetin and myricetin could resist indomethacin-induced barrier dysfunction in rat intestinal epithelial (IEC-6) cells [8]. In a cell model (HT-29 cells) of intestinal inflammation, it was found that luteolin efficiently could inhibit the secretion of these important cytokine-induced inflammatory mediators like interleukine-8 (IL-8), cyclooxygenase-2 (COX-2), and inducible nitric oxide synthase (iNOS), suggesting that this compound had the anti-inflammatory potential to alleviate intestinal inflammation [9]. In addition, a major component of tea polyphenols, epigallocatechin-3-gallate was observed to promote the immunological function of the intestine by inducing the secretion of anti-microbial peptides [10]. Due to their structural diversity, natural flavonoids (a family of polyphenol substances) are chemically divided into six major subgroups including flavones, flavonols, and others. To the best of our knowledge, whether the structural change of flavonols causes a changed anti-inflammatory potential on the IEC-6 cells is interesting but remains unclear so far.

Chemically, polyphenols have various numbers of hydroxyl groups in their skeleton structures, and these hydroxyl groups usually make the chemical properties of polyphenols extremely unstable. Thus, polyphenol stability is easily damaged by the changes in temperature, pH, oxygen, and other conditions [11]. It was reported that heat treatment of polyphenols led to changed fluorescence intensities and position of the spectra, indicating heat-induced polyphenol degradation [12]. During food processing, heat treatment is one of the most important strategies used for long-time food preservation. Thereby, heat treatment of plant foods can partly or completely inactivate enzymes and microorganisms, but also may impact the bioactivities of the polyphenols in these foods. It was found that heat treatment of two flavonols partly inhibited their activities to improve the barrier function of IEC-6 cells [13], while thermal treatment caused the white mulberry fruit extracts with weakened anti-oxidant potential but increased anti-cancer activity against HCT-116 cells [14]. Moreover, it was also found that the heat-treated eriodictyol had higher activity than the unheated one in the two immune cells in terms of splenocytes and macrophages [15]. However, whether heat treatment of natural flavonols (e.g., galangin and quercetin) might impact their anti-inflammatory function in the intestine is still not assessed. Such an investigation is thus essential to us.

**Figure 1:**
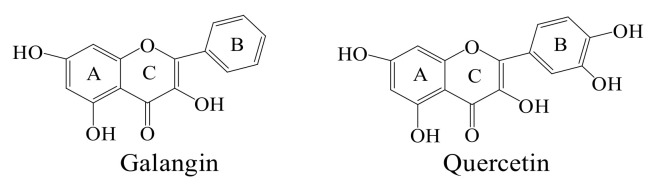
Chemical structures of two natural flavonols galangin and quercetin.

Using the LPS-induced IEC-6 cells as a cell model, this study assessed the anti-inflammatory activities of galangin and quercetin (with or without heat treatment) (Figure 1) towards rat intestinal epithelial (IEC-6) cells by measuring the production changes of three pro-inflammatory cytokines, two anti-inflammatory cytokines and another pro-inflammatory mediator prostaglandin E_2_ (PGE_2_). In addition, the changes of anti-inflammatory activity of the flavonols in response to the heat treatment at 100 °C for 15–30 min were verified. Moreover, whether the flavonols had a regulation on the inflammation-related NF-κB signaling pathway of IEC-6 cells was revealed by the Western-blot assays and then evidenced by theoretical calculation using the molecular docking analysis.

## 2. Results

### 2.1. Effect of the Flavonols on Growth of IEC-6 Cells

**Figure 2:**
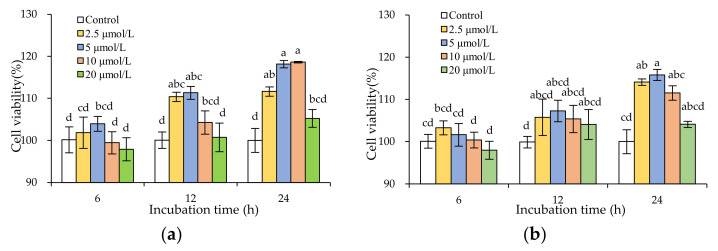
The measured viability values of the IEC-6 cells exposed to galangin (**a**) and quercetin (**b**) for treatment times of 6–24 h. Different lowercase letters (a–d) above the columns indicate that the mean values differ significantly (*p* < 0.05).

To avoid possible cytotoxicity of the two flavonols on IEC-6 cells, four flavonol dosages (2.5–20 μmol/L) were investigated in this study to verify whether the flavonols at these dosages might have an adverse effect on cell growth. When the cells were treated with galangin and quercetin at these dosages for three time periods (6, 12, and 24 h), the results from the classic MTT assay indicated that the treated cells had viability values of 97.9–118.6% (Figure 2). The flavonols with a shorter incubating time (6 h) for the cells sometimes caused a slight growth inhibition on the cells, yielding viability values slightly less than 100%. However, the flavonols with longer incubating times (i.e., 12 and 24 h) for the cells always exerted growth proliferation on the cells, leading to viability values larger than 100%. The flavonol dosage of 5 μmol/L yielded the highest viability values, while the flavonol dosage of 20 μmol/L showed a tendency to decrease viability values. Moreover, galangin mostly showed slightly higher activity to promote cell growth than quercetin (viability values 97.9–118.6% vs. 98.0–115.8%). All results confirmed that the flavonols at these evaluated dosages had an insignificant toxic effect on the cells, while the flavonols at 5 μmol/L could be used to evaluate their potential anti-inflammatory activities towards the LPS-stimulated IEC-6 cells.

### 2.2. Effect of the Flavonols on the Production of Pro-Inflammatory Cytokines and PGE_2_

To evaluate the anti-inflammatory activities of galangin and quercetin, as well as the impact of the heat treatment on flavonol activities, IEC-6 cells were treated with or without these flavonols (heated and unheated) for 1 h and subsequently stimulated by LPS for 24 h. The analysis results demonstrated that the three pro-inflammatory cytokines (IL-1β, IL-6 and TNF-α) and another mediator PGE_2_ in the medium showed a clear change in response to the performed LPS and flavonol treatments (Table 1). PGE_2_ level of the cells was used as an inflammatory indicator in this study, based on the fact that inflammation is multiple processes that also leads to a large release of PGE_2_ [16]. In brief, the control cells without LPS and flavonol treatments had the lowest levels of the four inflammatory mediators, while the model cells (treated with LPS only) showed the highest values for the four indices (*p* < 0.05). That is, LPS indeed caused inflammation in the cells. Meanwhile, compared with the model cells, the flavonol-treated cells mostly had a significant reduction in the values of the four indices (*p* < 0.05), declaring the flavonols could antagonize the LPS-induced inflammation in the cells efficiently. It is well-known that the BAY 11-7082 is an inhibitor of the NF-κB signaling pathway [17]. Thus, the inhibitor BAY 11-7082 in the cells was found to have the ability to inhibit IL-6 production (by decreasing its level from 205.7 to 102.3 pg/mL, Table 1), although its effect on the other three mediators was not assessed in this study. The cells with BAY 11-7082 treatment showed less inflammation than the model cells, suggesting that this inhibitor blocked the NF-κB signaling pathway and subsequently caused reduced inflammation. This finding also confirmed the anti-inflammatory potential of the two flavonols. Moreover, the results from a data comparison also indicated that galangin had higher anti-inflammatory activity in the cells to reduce the values of these inflammatory mediators than quercetin. In addition, the unheated flavonols showed higher anti-inflammatory effects than the heated counterparts (especially those with a heat time of 30 min) to reduce the values of the four inflammatory mediators. This fact means that heat treatment of the flavonols consistently reduced their capacity to combat against the LPS-induced inflammation in IEC-6 cells.

### 2.3. Effect of the Flavonols on the Production of Anti-inflammatory Cytokines

On the other hand, whether the flavonols could combat LPS-induced inflammation in IEC-6 cells was assessed via verifying the production changes of two anti-inflammatory mediators namely IL-10 and TGF-β. In response to the stimuli of LPS and these heated or unheated flavonols, the cells showed different production levels in IL-10 and TGF-β (Table 2). Compared with the control cells without LPS and flavonol treatments, the model cells with LPS treatment alone showed a suppressed IL-10 and TGF-β production, because their values were decreased from 26.1 and 36.8 to 6.5 and 29.4 pg/mL, respectively. The value decreases clearly reflected the LPS-caused cell inflammation. In response to the flavonol treatments, the cells after LPS stimulus showed an enhancement in the production of IL-10 (17.4–35.9 pg/mL) and TGF-β (32.0–45.6 pg/mL). That is, the flavonol-treated cells received higher production of IL-10 and TGF-β. In other words, the flavonols exerted an anti-inflammatory effect on the LPS-injured cells by promoting the production of the two anti-inflammatory cytokines. As expected, the inhibitor BAY 11-7082 with an ability to block the NF-κB signaling pathway also showed an ability to promote IL-10 level to 25.5 pg/mL (Table 2), and thereby proved the anti-inflammatory function of the flavonols in the cells. In addition, galangin in the cells was more efficient than quercetin to promote the production of IL-10 and TGF-β, while the heated flavonols (especially those heated for 30 min) consistently showed a weaker potential than the unheated counterparts in this case.

### 2.4. Effect of the Flavonols on the LPS-Induced Activation of NF-κB Signaling Pathway

**Figure 3:**
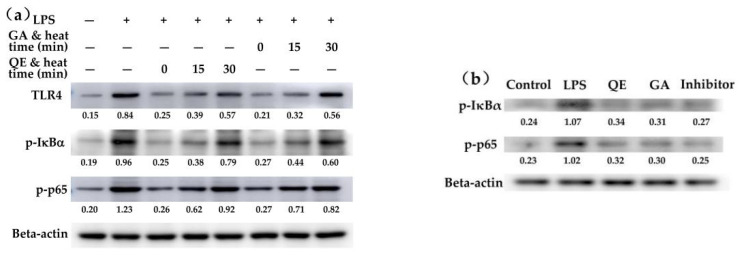
The anti-inflammatory effect of galangin and quercetin on the IEC-6 cells with LPS-caused inflammation.

It is well known that the NF-κB signaling pathway is a key pathway involved in both immune and anti-inflammatory functions [18]. To further reveal the target anti-inflammatory activities of the flavonols in the cells, three critical proteins involved in this signaling pathway, namely TLR4, p-IκBα and p-p65, were detected for their expression changes in the treated cells (Figure 3a). The results showed that the expression levels of TLR4, p-IκBα and p-p65 in the model cells were up-regulated in response to LPS stimulation, compared with these levels of TLR4, p-IκBα and p-p65 in the control cells without LPS stimulation. Thus, LPS activated NF-κB signaling pathway and subsequently induced inflammatory responses in the cells. Meanwhile, the flavonol-treated cells were consistently measured with less expression of TLR4 together with reduced levels of p-IκBα and p-p65, compared with the model cells. In addition, it was found that the up-regulated expression of p-IκBα and p-p65 in the cells was efficiently suppressed by the NF-κB inhibitor BAY 11-7082 (Figure 3b), providing further evidence that LPS induced inflammatory response in an NF-κB-dependent manner. The western-blot results thus disclosed that it was via an activation on the NF-κB signaling pathway that the flavonols exerted anti-inflammatory activities in the cells to attenuate the LPS-induced inflammatory responses (Figure 3). Furthermore, the heated flavonols (especially those with a heat time of 30 min) had a decreased potential versus the unheated counterparts to suppress TLR4/NF-κB activation, while galangin was more efficient than quercetin to suppress TLR4 expression (relative protein expression 0.21 vs. 0.25) (Figure 3a). However, whether galangin had a superior ability than quercetin in the cells to inhibit the expression of p-IκBα and p-p65 was unclear in the present assay, and thus needs a future investigation.

### 2.5. The Theoretical Affinity of the Flavonols to the Target TLR4/NF-κB

**Figure 4:**
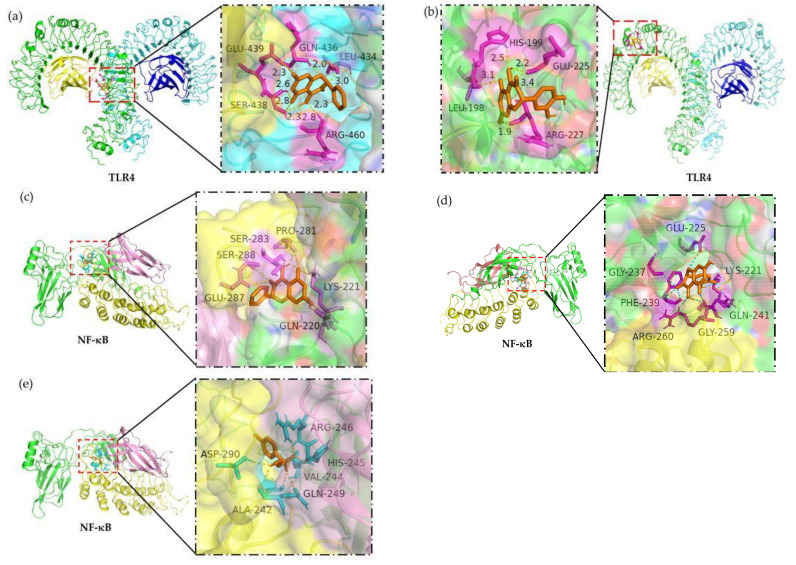
In silico analysis 3D results of the possible covalent interactions between the target TLR4/NF-κB proteins and the target molecules including galangin (**a,c**), quercetin (**b,d**), and the inhibitor BAY 11-7082 (**e**).

To obtain further evidence supporting the disclosed anti-inflammatory effects, molecular docking analysis was conducted. The obtained 3D results indicated that both galangin and quercetin could interact with target TLR4 and NF-κB at different regions, and thus were held in different pockets (Figure 4a–d). For the two flavonols with different chemical structures, the disclosed flavonol-TLR4 and flavonol-NF-κB interactions thereby involved in different amino acid residues in TLR4 or NF-κB (Table 3), which thus caused different interaction extents and energy changes.

**Figure 5:**
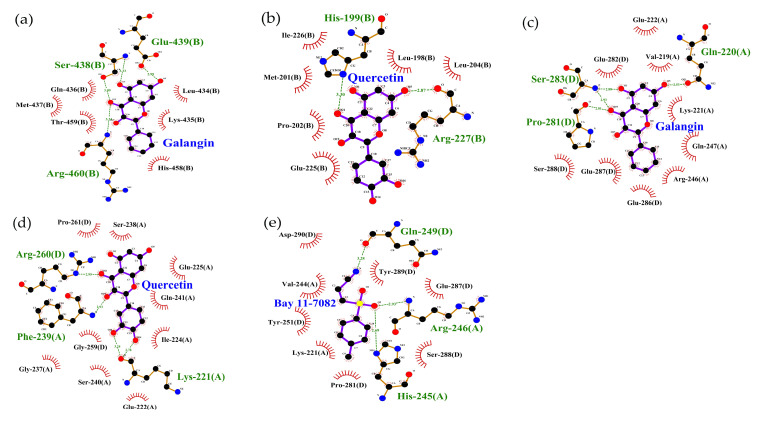
The 2D results for the chemical groups involved in the interactions between TLR4/NF-κB proteins and the target molecules including galangin (**a,c**), quercetin (**b, d**), and the inhibitor BAY 11-7082 (**e**).

In addition, the 2D model results revealed the chemical groups involved in the interactions between the flavonols and receptor proteins (Figure 5). For the target TLR4, the 7-OH group of galangin interacted with Glu-439 by forming a H-bond, while the 5-OH group also interacted with Ser-438 to form a 0.333 nm distance H-bond. Meanwhile, the 7-OH group of quercetin also formed a H-bond with Arg-227 of the target TLR4. For the NF-κB, the 5-OH and 7-OH groups of galangin interacted with NF-κB protein through the formation of two H-bonds with Ser-283 and Gln-220, while quercetin might form two H-bonds with NF-κB protein by the interactions between 3′-OH or 4′-OH groups and Lys-221 or Phe-239, respectively. Furthermore, BAY 11-7082 could form three H-bonds with NF-κB protein at Arg-246, His-245, and Gln-249. In total, the flavonols were able to form H-bonds with the two receptor proteins.

Overall, these in silico analysis results showed that galangin had a higher affinity for TLR4 and NF-κB than quercetin, resulting in higher decreases in the binding energy (–23.4 vs. −21.9 kJ/mol for TLR4, or −28.6 vs. −28.2 kJ/mol for NF-κB) (Table 3). This finding might explain why galangin had higher suppression than quercetin on the TLR4/NF-κB activation in the cells because galangin was more effective than quercetin to interact with TLR4 and NF-κB. The in silico results for the interaction between BAY 11-7082 and NF-κB also showed that BAY 11-7082 could interact with NF-κB strongly (Figure 5e), resulting in the highest decrease of binding energy (−29.0 kJ/mol, Table 3). Based on the results described above, it was reasonable that galangin had higher anti-inflammatory activity in the LPS-injured cells than quercetin.

## 3. Discussion

Under poor dietary conditions, the intestinal flora is usually in a disordered state. The accumulation of the adverse endogenous toxins and LPS continues to stimulate the colonic immune system and epithelial cells. A deteriorated immune system of the body may eventually lead to colonic inflammation [19,20]. Thus, alleviating the undesired colonic inflammation is vital to the body’s health. Fortunately, the previous results had revealed that a variety of food components could suppress intestinal inflammation and thus were regarded capable of maintaining the health of the body; for example, the peptide fractions from the dehydrated potatoes possessed anti-inflammatory activity to IEC-6 cells [21], while the polysaccharide from Schizophyllum commune could recover the DSS-induced colitis in the inflamed tissues, and thus showed an anti-inflammatory effect on the intestine [22]. It was also found that a heteropolysaccharide namely SHPS-1 (isolated from fruiting bodies of Phellinus baumii) showed a capacity to alleviate ulcerative colitis in mice model by decreasing the pro-inflammatory genes and increasing the anti-inflammatory and tissue repairing genes [23]. In addition, polyphenols were also regarded to have an anti-inflammatory capacity; for example, the extracts from grape seed and grape marc meal could inhibit the pro-inflammatory NF-κB signaling pathway in intestinal epithelial cells [24], while those from red wine extract had an inhibitory effect on IL-8 production as well as COX-2 and iNOS induction and then ameliorated or prevented intestinal inflammation [25]. In the present study, the results demonstrated that the flavonols could exert anti-inflammatory activities against the LPS-induced inflammation in IEC-6 cells, via increasing the production of two anti-inflammatory mediators and decreasing the production of four inflammatory mediators. The present study thus shared a conclusion in consistence with these previous studies [24,25,26,27].

To verify scientifically how the flavonols exerted an anti-inflammatory effect on the LPS-stimulated IEC-6 cells, possible signaling pathways involved in this effect thereby need an investigation. It has been well-established that the release of inflammatory and pro-inflammatory mediators during an inflammatory response in cells is regulated by inflammatory signaling pathways [28]. For example, the NF-κB translocation into the nucleus can mediate the rate of IL-10 transcription and expression of TGF-β [29,30,31]. A variety of food components can affect these signaling pathways and subsequently exert their anti-inflammatory activities. It was evident that the anti-inflammatory effect of the PJ-1 polysaccharide in macrophages depended on the activation of the important signal transducers and activators of transcription 3 (STAT3) pathways [32], while the glycosides from Tripterygium wilfordii could promote the production of IL-37 (an anti-inflammatory cytokine) in THP-1 cells [33]. Moreover, both ERK1/2 and p38 MAPK signaling pathways were involved in the assessed anti-inflammatory effect of the glycosides [33]. TLR4/NF-κB signaling pathway, as a classic implant-related innate immune recognition and key immuno-modulation signaling pathway, has attracted widespread attention [34]. LPS has been classified as an activator of the TLR4/NF-κB signaling pathway [35,36]. Two previous studies thus indicated that LPS could induce cell inflammation by an up-regulated expression of p-IKKα, p-IKKβ, and p-IκBα in the NF-κB signaling pathway [37,38]. In this study, BAY 11-7082 as an inhibitor of the NF-κB signaling pathway exerted a clear down-expression on p-IκBα and p-p65 in the cells. Thus, BAY 11-7082 exposure of the cells reasonably resulted in a blocked NF-κB signaling pathway and subsequently an increased IL-10 but decreased IL-6 levels. Meanwhile, the western-blot assay results proved a down-regulated protein expression of TLR4, p-IκBα and p-p65 by the flavonols. Thus, the flavonols were regarded to exhibit anti-inflammatory activities to the cells via suppression of the NF-κB signaling pathway. The present conclusion was in agreement with that obtained from two previous studies [39,40], in which a polyphenol compound iso-liquiritigenin and a nitrogen-containing compound tetra-methylpyrazine exerted anti-inflammatory effect via the suppression of the NF-κB signaling pathway. In addition, it was reported in three studies that polyphenols could suppress the activation of the NF-κB signaling pathway [41,42,43], which also provided consistent support to the present study. Overall, the western-blot results thus disclosed that it was via inhibition on the activation of the NF-κB signaling pathway that the flavonols exerted a clear anti-inflammatory effect on the cells to attenuate the LPS-induced inflammatory responses (Figure 3).

To further reveal the possible reason why the flavonols could suppress the activation of the NF-κB signaling pathway in the LPS-stimulated IEC-6 cells, molecular docking analysis was employed to uncover possible interactions between critical TLR4/NF-κB and the flavonols. Using this theoretical analysis, ginsenoside Rb1 was regarded to be held in the pocket of the TLR4-MD-2 complex by interaction with multiple amino acid residues [44], while epigallocatechin gallate was considered to have a good binding profile with the binding sites of IKK-α, IKK-β and NIK [45]. More importantly, the pyrazole derivatives were regarded to fit into the inner grove of the active site of NF-ĸB, resulting in an inhibition of the NF-ĸB signaling pathway [46]. In this study, the theoretical calculation results suggested potential interactions between TLR4/NF-κB and the flavonols and pointed out that galangin caused greater interaction (or higher energy decrease) than quercetin, which thus suggested that galangin might have higher suppression on the NF-κB signaling pathway and subsequently exerted a higher anti-inflammatory activity than quercetin. Additionally, the molecular docking results for the interaction between NF-κB and its inhibitor BAY 11-7082 provided support to the target flavonol-TLR4 and flavonol-NF-κB interactions, because the BAY 11-7082 caused an energy decrease similar to the flavonols. The OH groups are the bioactive function of flavonoids, while their roles for the target bioactivity had been identified. For example, it was revealed that the 3-OH but not 5-OH of the flavonoids was critical to the inhibition of arginase [47], while the 3-OH, 7-OH as well as OH in the B-ring of flavonoids involved in the H-bond binding with α-glycosidase [48]. Moreover, the 2D results also demonstrated that resveratrol could bind with IκB-alpha at Glu-49, Arg-50, and His-181 of the p65 to induce the apoptosis of U937 tumor cells [49], and the formation of H-bond interaction between the OH group of ferulic acid and TLR4 could combat against the LPS-induced neuroinflammation in the mouse brain [50]. Thus, it was confirmed in this study that the two flavonols could bind with these amino acid residues of two receptor proteins, causing H-bond formation and energy decrease. Hence, the obtained in silico results were consistent with these cell results, demonstrating again that it was through a suppression on the TLR4/NF-κB activation that the flavonols exerted anti-inflammatory activities in the cells to alleviate the LPS-induced inflammation.

Before human consumption, plant foods are usually heated via boiling, frying, griddling, stewing, or sterilizing treatments. These heat treatments can induce an alteration in component contents and more importantly their bioactivities. It was revealed that a polyphenol compound catechin in green tea would undergo various reactions during its heat-drying processing, including degradation, polymerization and isomerization [51]. Moreover, past studies had found that cooking temperature strongly affected proliferative inhibition of vegetable juices on HL-60 cells [52], while thermal processing of haskap berry had an impact on its bioactivities due to the cyanidin-3-O-glucoside degradation [53]. Additionally, it was verified that fresh mushrooms had higher activity than the processed ones, due to the chemical susceptibility of the anti-inflammatory compounds to the used heat treatment [54]. It is thus reasonable in this study that the heated flavonols had weaker anti-inflammatory effects than the unheated ones on the LPS-injured cells. Based on assay results of the mass spectra, it was verified that heat treatment of catechin under alkaline conditions triggered cleavage of the heterocyclic ring (i.e., the C-ring) via the oxidation of the ether bond [55]. After thermal treatment, quercetin yielded the formation of various degraded substances like 2,4,6-trihydroxymandelate and 2,4,6-trihydroxyphenylgloxylate [56]. It was also found that higher temperature endowed the isomerized polyphenols with lower bioactivity [57]. Thus, it is necessary in the future to investigate why the heated flavonols had an impaired activity in the cells.

Chemically, different skeleton structures of polyphenols might yield different bioactivities. Previous results had verified that the presence of a catechol group in the B-ring, 3-OH group, and C2-C3 double bond was critical to the anti-oxidative capacity of flavonoids [58]. The OH group positions, double bonds, and B-ring structure thus seem to be influential for the anti-inflammatory activity of flavonoids [59]. It was reasonable that galangin and quercetin were measured with different anti-inflammatory activity in the LPS-injured cells, due to their different chemical structures. It has been suggested that the OH groups are vital for the anti-inflammatory function of flavones; for example, an OH group at C-3′ might attenuate their bioactivity [60]. Galangin is only different from quercetin in the B-ring (no OH group versus two OH groups, Figure 1). The less hydroxyl group flavonol contains, the smaller polarity of the compound is, and thereby the compound can interact with cell membrane easily [61]. Galangin with fewer OH groups in its B-ring, while quercetin has two OH groups in its B-ring and especially a OH group at C-3′ that can attenuate its anti-inflammatory activity [60]. In addition, it had been proposed that flavonoids with less non-modification in the B-ring had the greatest interactivity with the cell membrane [61]. Thus, galangin had higher anti-inflammatory potential than quercetin in the cells.

## 4. Materials and Methods

### 4.1. Reagents and Materials

Both galangin and quercetin (declared purity > 97%) were bought from Dalian Meilun Biological Technology Co. Ltd. (Dalian, Liaoning, China). The LPS (L2880), Dulbecco’s modified Eagle’s medium (DMEM) (D5648), and 3-(4,5-di-methylthiazol-2-yl)-2,5- diphenyltetrazolium bromide (MTT) (M5655) were all purchased from Sigma-Aldrich Co. (St Louis, MO, USA). Fetal bovine serum (FBS) was provided by Wisent Inc (Montreal, QC, Canada). Bovine insulin, DMSO, phosphate-buffered saline (PBS), and trypsin-EDTA were all purchased from Solarbio Science and Technology Co., Ltd. (Beijing, China). All Enzyme-Linked Immuno Sorbent Assay (ELISA) kits were bought from Nanjing Jiancheng Bioengineering Institute (Nanjing, Jiangsu, China). BAY 11-7082 (a NF-κB inhibitor), radioimmunoprecipitation assay (RIPA) lysis buffer and BCA protein assay kit were purchased from Beyotime Institute of Biotechnology (Shanghai, China). The primary antibodies used for phosphor-NF-κB p65 (p-p65) (Bioss bs-0982R), TLR4 (Bioss bs-20594R), and β-actin (Bioss bs-0061R) were all provided by Biosynthesis Biotechnology Inc. (Beijing, China). The primary antibodies of phosphor-IκB alpha (p-IκBα) (CST 2859) and the goat anti-rabbit horseradish peroxidase (HRP) secondary antibody (CST 7074) were obtained from Cell Signaling Technology (Danvers, MA, USA). Other chemicals used were analytical grade. Ultrapure water was generated by Milli-Q Plus system (Millipore Corporation, New York, NY, USA) and used in this study.

### 4.2. Sample Preparation

Galangin (or quercetin) was dissolved in DMSO to reach a concentration of 20 mmol/L and diluted with serum-free medium to four dosage levels of 2.5–20 μmol/L with final DMSO concentration less than 0.1%. Afterward, the solution of each flavonol dosage was divided into two parts: one part with serum addition for cell experiments directly, another part with heat treatment (100 °C for 15–30 min followed by ice cooling) and serum addition for cell experiments immediately.

### 4.3. Cell Culture and MTT Assay

IEC-6 cells that have the characteristics of the stable passage of crypt epithelial cells were obtained from the American Type Culture Collection (Rockville, MD, USA). As recommended, the cells were cultured in the DMEM containing 10% FBS, 1% sodium pyruvate, 100 U/L bovine insulin, and 100 mg/mL penicillin/streptomycin. Incubator temperature was set at 37 °C together with a humidified environment containing 5% CO_2_. The same protocol was used in most cell experiments; that is, the cells were starved in a serum-free medium for 12 h and then pre-treated with the flavonols for 1 h. After the medium was replaced, the cells were exposed to LPS alone for 24 h.

The cells (2 × 10^3^ cells/well) were inoculated in 96-well plates. When the cells were attached, they were starved in serum-free medium for 12 h. Flavonol solutions of 100 μL were added to each well, while the cells were incubated at 37 °C for 6, 12, and 24 h, respectively. After medium removal, the cells were washed three times with PBS and incubated with 100 µL MTT reagent (1 mg/mL) for 4 h. After discarding medium, 150 μL DMSO was added into each well, while the plates were shaken gently for 10 min. The absorbance value of each well was detected at 490 nm using a microplate reader (Bio-Rad Laboratories, Hercules, CA, USA). Cell viability was calculated as previously described [62]. The control cells without flavonol exposure were designed with a viability value of 100%.

### 4.4. Assays of Cytokines and PGE_2_ Production

The levels of the inflammation-related cytokines and PGE_2_ were assessed using respective ELISA kits. IEC-6 cells were inoculated into 24-well plates (8 × 10^4^ cells/well), allowed to adhere for 24 h, treated in the serum-free medium for 12 h, incubated with the flavonols at 5 μmol/L (or BAY 11-7082 at 10 μmol/L) for 1 h, and then exposed to LPS for 24 h. The supernatants were collected and then measured for the levels of five cytokines and PGE_2_ using the protocols provided by the kit manufacturers. The target pro-inflammatory cytokines were IL-1β, IL-6 and tumor necrosis factor-alpha (TNF-α), while the measured anti-inflammatory cytokines were IL-10 and transforming growth factor-beta (TGF-β).

### 4.5. Western-Blot Assay

The cells were treated by flavonols or BAY 11-7082 as above, whereas the cell homogenates were obtained at 30 min after LPS stimulation as described previously [36]. The cells were washed with PBS at 4 °C for three times. RIPA cleavage buffer and PMSF (Beyotime, Shanghai, China) were used to lyse the cells on ice for 30 min, followed by centrifugation at 12,000× *g* for 5 min at 4 °C to extract total protein. Quantitative determination of total protein was performed using the BCA protein analysis kit. The extracted proteins were separated using 4–12% SDS-PAGE and transferred to PVDF membranes, which were placed in 5% skim milk in PBS containing 0.1% Tween-20 (i.e., PBST) and sealed at 37 °C for 120 min. Primary antibody β-actin (1:10,000 dilution), TLR4, p-IκBα and p-p65 (1:1000 dilution for each) were used and kept overnight at 4 °C. After washing three times with PBST, the membrane was incubated with the HRP-conjugated secondary antibody (1:2000 dilution) at 37 °C for 1 h and then visualized with enhanced chemiluminescence reagent (Biosharp Life Sciences, China) after washing three times with TBST for 10 min each time. Moreover, the ChemiDoc^™^ MP Imaging System (Bio-Rad Laboratories, Hercules, CA, USA) were used to detect protein bands, while the Image J software (National Institutes of Health, Bethesda, MD, USA) was used in quantitative analysis. The band density was normalized to an endogenous reference β-actin.

### 4.6. Molecular Docking Analysis

The crystal structures of TLR4 (PDB code, 3FXI) and NF-κB (PDB code, 1IKN) were downloaded from the RCSB Protein Data Bank (https://www.rcsb.org/pdb) (accessed on 10 July 2021). The three-dimensional structures of galangin and quercetin were obtained from the ZINC15 database (http://zinc15.docking.org) accessed on 3 August 2021), while that of the inhibitor BAY 11-7082 was provided by the PubChem database (https://pubchem.ncbi.nlm.nih.gov) (accessed on 3 August 2021). The PyMOL Molecular Graphics System (PyMOL, Version 2.4.0, Schrödinger, LLC, New York, NY, USA) and AutoDockTools (Version 1.5.6, Molecular Graphics Laboratory, The Scripps Research Institute, San Diego, CA, USA) were used for the ligand-receptor docking as previously described [63]. The grid size, which covered the whole protein, was set to 11.0, 11.0 and 11.0 nm (x, y, and z) with the spaces of 0.1000 and 0.0508 nm for TLR4 and NF-κB, respectively. In this assay, the binding affinity of the flavonols or inhibitor to TLR4 and NF-κB was regarded to yield the highest energy decrease.

### 4.7. Statistical Analysis

All experiments or assays were performed at least three times, while the data are reported as the means or means ± standard deviations. One-way analysis of variance (ANOVA) was used for the significance analysis among different groups (*p <* 0.05) using the Social Science Statistical Program 16.0 software package (SPSS Inc., Chicago, IL, USA) and Duncan’s multiple comparisons.

## 5. Conclusions

Both galangin and quercetin had anti-inflammatory activities towards the LPS-stimulated IEC-6 cells, leading to the suppressed release of four pro-inflammatory mediators (PGE_2_, IL-1β, IL-6 and TNF-α) and enhanced production of two anti-inflammatory mediators (IL-10 and TGF-β). Anti-inflammatory effect of the two flavonols on the stimulated cells was achieved by suppressing the TLR4/NF-κB signaling pathway, through a down-regulation on the critical TLR4, p-IκBα and p-p65; moreover, the in silico analysis results using molecular docking revealed potential flavonol-TLR4 and flavonol-NF-κB interactions, supporting the suppressed TLR4/NF-κB signaling pathway. In addition, the results using an NF-κB inhibitor BAY 11-7082 also provided consistent support for these mentioned results. The two flavonols thus were regarded to have a beneficial effect on the body and might be potential candidates to alleviate the adverse intestinal inflammation. Galangin had higher anti-inflammatory potential than quercetin, due to different chemical features in the B-ring. However, the applied heat treatment (especially that using longer heat time) consistently caused decreased anti-inflammatory activities for the two flavonols in the cells.

## Figures and Tables

**Table 1:** Detected levels (pg/mL) of three pro-inflammatory cytokines and PGE_2_ in IEC-6 cells with or without LPS, flavonols, and inhibitor (BAY 11-7082) treatments.

Cell Group ^1^	IL-1β	IL-6	TNF-α	PGE_2_
Control	6.0 ± 0.3 ^f^	88.0 ± 2.7 ^f^	52.0 ± 4.6 ^e^	5.1 ± 0.5 ^d^
Model	40.5 ± 4.0 ^a^	205.7 ± 13.4 ^a^	206.3 ± 12.2 ^a^	19.3 ± 1.7 ^a^
Galangin	23.4 ± 2.1 ^e^	129.7 ± 10.6 ^d^	147.3 ± 4.9 ^d^	5.8 ± 0.6 ^d^
Heated GA-15	29.2 ± 1.7 ^c,d^	133.1 ± 6.1 ^d^	156.3 ± 4.2 ^c,d^	6.9 ± 0.7 ^b,c,d^
Heated GA-30	31.8 ± 1.2 ^b,c^	152.9 ± 6.8 ^b,c^	172.3 ± 6.1 ^b,c^	8.3 ± 0.3 ^b,c^
Quercetin	26.3 ± 3.3 ^d,e^	133.0 ± 7.6 ^d^	154.0 ± 10.5 ^d^	6.1 ± 1.1 ^c,d^
Heated QE-15	32.6 ± 3.2 ^b,c^	139.4 ± 3.1 ^c,d^	163.0 ± 8.0 ^b,c,d^	7.2 ± 1.0 ^b,c,d^
Heated QE-30	36.2 ± 1.8 ^a,b^	159.2 ± 8.1 ^b^	177. 7 ± 7.0 ^b^	8.7 ± 0.2 ^b^
Inhibitor	Not assessed	102.3 ± 7.0 ^e^	Not assessed	Not assessed

^1^ GA, galangin; QE, quercetin; the numbers following these flavonol names indicate the heat time (min) used in the sample preparation. Different lowercase letters (a–f) after the data as the superscripts in the same column indicate that the mean values of ANOVA using Duncan’s multiple comparison test differ significantly (*p* < 0.05).

**Table 2:** Detected levels (pg/mL) of two anti-inflammatory cytokines in IEC-6 cells with or without LPS, flavonols, and inhibitor (BAY 11-7082) treatments.

Cell Group ^1^	IL-10	TGF-β
Control	26.1 ± 1.4 ^c,d^	36.8 ± 1.7 ^b,c,d^
Model	6.5 ± 1.3 ^f^	29.4 ± 1.6 ^f^
Galangin	35.9 ± 1.4 ^a^	45.6 ± 1.1 ^a^
Heated GA-15	32.8 ± 1.4 ^b^	41.0 ± 2.5 ^a,b^
Heated GA-30	28.2 ± 1.2 ^c^	34.1 ± 3.1 ^d,e^
Quercetin	27.4 ± 1.2 ^c^	40.5 ± 3.5 ^b,c^
Heated QE-15	24.2 ± 0.7 ^d^	36.1 ± 1.4 ^c,d,e^
Heated QE-30	17.4 ± 2.2^e^	32.0 ± 2.2 ^e,f^
Inhibitor	25.5 ± 1.0 ^c,d^	Not assessed

^1^ GA, galangin; QE, quercetin; the numbers following these flavonol names indicate the heat time (min) used in the sample preparation. Different lowercase letters (a–f) after the data as the superscripts in the same column indicate that the mean values of ANOVA using Duncan’s multiple comparison test differ significantly (*p <* 0.05).

**Table 3:** Analysis results for the affinity of two flavonols or inhibitor (BAY 11-7082) to TLR4 or NF-κB.

Binding Model	Binding Energy (kJ/mol)	Amino Acid Residues Involved in the Interaction
Galangin-TLR4	−23.4	Leu-434, Lys-435, Gln-436, Met-437, Ser-438 *, Glu-439 *, His-458, Thr-459, Arg-460 *
Quercetin-TLR4	−21.9	Leu-198, His-199*, Met-201, Pro-202, Leu-204, Glu-225, Ile-226, Arg-227*
Galangin-NF-κB	−28.6	Val-219, Gln-220 *, Lys-221, Glu-222, Arg-246, Gln-247, Pro-281 *, Glu-282, Ser-283 *, Glu-286, Glu-287, Ser-288
Quercetin-NF-κB	−28.2	Lys-221 *, Glu-222, Ile-224, Glu-225, Gly-237, Ser-238, Ser-240, Phe-239 *, Gln-241, Gly-259, Arg-260 *, Pro-261
Inhibitor-NF-κB	−29.0	Lys-221, Val-244, His-245 *, Tyr251, Arg-246 *, Gln-249 *, Pro-281, Glu-287, Ser-288, Tyr-289, Asp-290

The residues with asterisks indicate H-bonding with these residues.

## Data Availability

All data are contained within the article.

## Sample Availability

Samples of the compounds galangin, quercetin and BAY 11-7082 are available from the authors.
